# Abdominal pain and fever in a patient with human immunodeficiency virus: a case report

**DOI:** 10.1186/s12245-022-00439-z

**Published:** 2022-08-08

**Authors:** Max Ackerman, Arian Frost, Kimberly Stanford

**Affiliations:** 1 University of Chicago Pritzker School of Medicine, 924 E 57th St #104, Chicago, IL 60637 USA; 2 Department of Medicine, Section of Emergency Medicine, University of Chicago, 5841 S Maryland Ave, Chicago, IL 60637 USA

**Keywords:** Neutropenic enterocolitis, Typhlitis, Human immunodeficiency virus, Neutropenia

## Abstract

**Background:**

Neutropenic enterocolitis (NE), or typhlitis, a condition typically associated with severe neutropenia in the setting of chemotherapy, is highly morbid (50–100%) and benefits from early diagnosis. It has been associated with neutropenia in the setting of human immunodeficiency virus (HIV) but has not been described in a patient with HIV who was not neutropenic on presentation. We present the case of a patient with HIV who was not neutropenic on presentation but found to have NE.

**Case presentation:**

A 27-year-old male with a history of HIV on antiretroviral therapy and epilepsy presented with concern for breakthrough seizure. The patient revealed he was having non-bloody, non-bilious emesis and diarrhea for 3 days. Initial labs were white blood cell count 3.9 × 10^9^/L, absolute neutrophil count (ANC) 3.14 × 10^9^/L, CD4 count 290 cells/mm^3^, and undetectable viral load. A computed tomography (CT) scan of the abdomen/pelvis with contrast revealed wall thickening of the cecum and proximal ascending colon (Fig. 1), suggestive of NE. The patient was started on cefepime and metronidazole but switched to piperacillin/tazobactam after he became leukopenic/neutropenic.

**Conclusions:**

Neutropenic enterocolitis, typically presenting with fever, abdominal pain, and hematochezia, can be difficult to identify, particularly in patients without a history of malignancy. However, it should be considered in patients with HIV presenting with these symptoms, even with a normal ANC and CD4 count above 200 cells/mm^3^. Prompt diagnosis can be made with CT, and early initiation of broad-spectrum antibiotics greatly reduces the risk of morbidity/mortality.

## Background

Neutropenic enterocolitis (NE), also referred to as typhlitis or ileocecal syndrome, is a rare but potentially life-threatening condition occurring primarily in neutropenic patients [1]. Neutropenic enterocolitis is characterized by severe inflammation of the ileocecal region, but other parts of the colon may be involved as well. It typically presents with fever, abdominal pain, abdominal distention, and diarrhea, and the severe intestinal inflammation can cause bowel ischemia resulting in necrosis and bowel perforation [2].

Neutropenic enterocolitis may occur in children or adults, and it commonly presents during periods of severe neutropenia after administration of high-dose chemotherapy for hematologic malignancies (e.g., leukemia, lymphoma, multiple myeloma). It has more rarely been associated with other etiologies of immunosuppression such as human immunodeficiency virus (HIV), organ transplant, and therapy for solid tumors [3]. The main cause of death in patients with NE is septicemia caused by bacterial or fungal translocation across the intestinal wall [4]. The mortality rate of patients with NE has been reported to be between 50% and 100%, and it increases when treatment is delayed [5]. In such a highly morbid condition, it is important that emergency medicine clinicians are aware of the diagnosis and treatment of NE, in addition to atypical presentations of the disease.

To the best of our knowledge, NE has not been described previously in a patient with HIV who was not neutropenic on presentation to the emergency department (ED). In this report, we present the case of a male patient with a history of HIV who presented to the ED for abdominal pain and diarrhea, and on computed tomography (CT) imaging was found to have NE.

## Case presentation

A 27-year-old male with a history of HIV on antiretroviral therapy, asthma, alcohol use disorder, and epilepsy, presented to the ED with concern for a breakthrough seizure. He was at home with his mother when his legs became rigid, and he was unable to move them. He reported good recollection of the entire event, no loss of consciousness, and spontaneous resolution after several minutes. His mother witnessed the event and called for an ambulance. The patient reported that his typical seizures are generalized, tonic-clonic in nature, with loss of consciousness. His last typical seizure occurred 1.5 months prior to presentation.

On further review of systems, the patient revealed he had been having nausea, non-bloody, non-bilious emesis, and diarrhea for 3 days. He had not been tolerating oral intake and had poor compliance with his antiepileptic medication due to nausea and vomiting. He reported multiple episodes of loose stool but denied frank blood or melena. He complained of generalized, constant abdominal pain without specific character or location. He also described tactile fever, headache, and night sweats over the preceding 3 days. The patient reported drinking a pint of liquor daily, but due to nausea and vomiting had not had any alcohol in 3 days.

Examination in the ED revealed a well appearing male in no distress. Vital signs were reassuring; the patient was afebrile (97 ^o^F) with a heart rate of 79 beats per minute and a blood pressure of 147/91 mm of mercury. Abdominal examination was notable for normal bowel sounds, and diffuse tenderness with voluntary guarding but no rebound. Neurologic examination was normal, and the patient had a normal mental status.

Initial finger stick glucose in the ED was 54 mg/dL (reference range 70–99 mg/dL), corrected to 124 mg/dL after an ampule of dextrose 50% intravenous (IV) push. Additional laboratory evaluation was notable for white blood cell count (WBC) 3.9 times 10^9^ cells per liter (10^9^/L) (reference range 3.5–11 × 10^9^/L), hemoglobin 10.6 g/dL (reference range 13.5–17.5 g/dL), neutrophils 80% (reference range 39–75%), and absolute neutrophil count (ANC) 3.14 times 10^9^ cells per liter (10^9^/L) (reference range 1.12–6.72 × 10^9^/L). Initial CD4 count was 290 cells in a cubic millimeter (cells/mm^3^) (reference range 500–1200 cells/mm^3^), and viral load was undetectable. Chemistry was notable for a metabolic acidosis with bicarbonate of 12 mEq/L (reference range 23–30 mEq/L) and anion gap 40 mmol/L (reference range 6–15 mmol/L) with a lactic acid of 5.0 mmol/L (reference range 0.5–2.2 mmol/L). Hepatic function panel was notable for elevated liver enzymes, with aspartate aminotransferase (AST) 2363 U/L (reference range 6–37 U/L) and alanine transaminase (ALT) 583 U/L (reference range 5–35 U/L). Acute hepatitis A, B, and C panel was not reactive, acetaminophen assay was negative, blood cultures were negative for growth after 5 days, and a stool panel testing for more than 20 common diarrheal pathogens, including C. difficile, was negative.

**Fig. 1:**
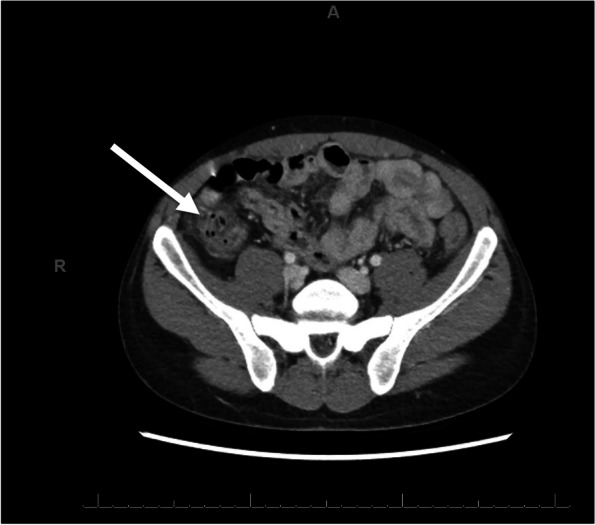
This figure shows an axial computed tomography scan with contrast of the abdomen of a patient with human immunodeficiency virus. The white arrow indicates mild wall thickening of the cecum and proximal ascending colon suggestive of neutropenic enterocolitis

A right upper quadrant ultrasound showed hepatomegaly and echogenic liver, favored to represent fatty infiltration in this clinical picture. No evidence of cholelithiasis or cholecystitis was seen. A CT scan of the abdomen and pelvis with contrast revealed wall thickening of the cecum and proximal ascending colon (Fig. 1), suggestive of NE.

The patient was initiated on cefepime (2 g IV every 8 h (Q8H)) and metronidazole (500 mg IV Q8H) and admitted for further management. During his admission, he became increasingly leukopenic and neutropenic, with a nadir of a total WBC of 1.6 × 10^9^/uL and ANC of 0.72 × 10^9^/uL 4 days after presentation to the ED. As a result, antibiotics were switched to piperacillin/tazobactam (4.5 g Q8H) until discharge. During his hospitalization he had several episodes of blood in his stool, a characteristic symptom of NE. Colonoscopy with histologic examination of the bowel mucosa for definitive diagnosis was deferred due to neutropenia and risk of perforation. His symptoms continued to improve, and he was successfully managed with bowel rest and antibiotics, with no operative intervention required. He was ultimately discharged on amoxicillin/clavulanic acid and trimethoprim/sulfamethoxazole for a total of 10 days of antibiotic coverage. His elevated liver enzymes were attributed to alcohol ingestion and improved during hospitalization, with an AST 450 U/L and ALT 309 U/L on discharge. Last known absolute CD4 count during admission was 141 cells/mm^3^ and his HIV-1 viral load was not detectable. While follow up is limited to presentations within our medical system, the patient did return to the ED 8 months after the initial presentation with mild rectal bleeding due to hemorrhoids and no mention was made of further episodes or complications of his NE.

## Discussion and conclusions

Neutropenic enterocolitis is a well-documented complication of chemotherapy for hematologic malignancies; however, the association between NE and HIV is far less reported. The pathogenesis of NE is not completely understood, and it may differ in patients being treated with chemotherapy versus those with HIV. In cancer patients, it appears that cytotoxic drugs may cause intestinal mucosal injury and profound neutropenia, leading to impaired host defense from intestinal flora. This microbial infection leads to production of bacterial endotoxins, bowel wall necrosis, hemorrhage, and subsequent septicemia. NE most often affects the cecum, and this may be due to its limited blood supply [6]. In patients who are not as profoundly neutropenic, such as those with HIV, a different pathogenesis may be at play. Patients infected with HIV have lost their cell-mediated immunity and thus may be more susceptible to opportunistic infections and malignancies of the intestinal mucosa. This mucosal injury also impairs the host defense against intestinal flora and pathogenic bacteria [7].

The incidence of NE among adults with hematologic malignancies varies greatly in the literature, ranging from 0.8 to 26%, but the incidence in patients with HIV is unexplored [8, 9]. Further, several authors report mortality rates of 50% or higher in patients with NE, and mortality rates are even greater when treatment is delayed [10]. Thus, prompt recognition of NE and initiation of treatment is crucial in reducing mortality. Patients classically present with a triad of symptoms: fever, abdominal pain, and neutropenia [2]. Localized pain to the right lower quadrant, abdominal distension, and diarrhea, sometimes with lower gastrointestinal (GI) bleeding, as was seen in this case, are likely to be present. A presentation with localized pain to the right lower quadrant can mimic acute appendicitis, while diarrhea and acute lower GI bleeding are more suggestive of NE [11]. Abdominal CT scan with contrast is the preferred diagnostic tool to distinguish between the two pathologies and identify bowel wall thickening (most often of the ileocecal region), which is the most common radiographic finding in NE [10]. Other CT findings in neutropenic patients may include pneumatosis intestinalis, mesenteric stranding, bowel dilation, and mucosal enhancement [12]. Ultrasound and plain radiographs may also be used, but these may be more likely to produce false negative results (23% and 48%, respectively) [12]. Ultimately, the gold standard for diagnosis of NE is histologic examination via colonoscopy [13]. However, this procedure is often contraindicated due to the risk of bowel perforation, as in this case.

Conservative management is the preferred approach for patients with NE. Upon recognition of the clinical and diagnostic indications for NE in patients with HIV, even without neutropenia, broad spectrum antimicrobial therapy should be initiated immediately [14]. Bowel rest, nasogastric suction, IV fluids, and nutritional support are also appropriate supportive treatments. Serial abdominal examinations should be conducted to monitor for deterioration of clinical condition, and clinicians should be aware of the potential for acute hemodynamic decompensation, especially if prompt management is not initiated [14]. Colectomy should be considered in case of acute hemodynamic instability, bowel perforation, massive hemorrhage, abscess formation, or lack of response to medical therapy [14]. Finally, with regard to the risk of recurrence, a study of eight patients with leukemia found the recurrence rate of NE after conservative management was 67% [15]. However, there have been no studies regarding the recurrence rate of NE in patients with HIV, so it is difficult to quantify in this specific population.

Overall, neutropenic enterocolitis, which typically presents with fever, abdominal pain, and bloody diarrhea, can be difficult to identify, particularly in patients without a history of malignancy. However, it should be considered in patients with HIV who present with these symptoms, even with a normal ANC and CD4 count above 200 cells/mm^3^. Prompt diagnosis can be made with CT imaging of the abdomen, and early initiation of broad-spectrum antibiotic therapy can greatly reduce the risk of morbidity and mortality in these patients.

## Data Availability

Data sharing is not applicable to this article as no datasets were generated or analyzed during the current study.

## Abbreviations

NE: Neutropenic enterocolitis
HIV: Human immunodeficiency virus
HAART: Highly active antiretroviral therapy
ED: Emergency department
CT: Computed tomography scan
EM: Emergency medicine
GI: Gastrointestinal
IV: Intravenous
Q8H: Every 8 h
mg/dL: Milligrams per deciliter
10^9^/L: 10^9^ cells per liter
cells/mm^3^: Cells in a cubic millimeter
U/L: Units per liter
AST: Aspartate aminotransferase
ALT: Alanine transaminase
